# Supplementary material for: Stress Buffering and Longevity Effects of Amber Extract on Caenorhabditis elegans (C. elegans)
Source: Molecules. 2022 Jun 16;27(12):3858. doi: 10.3390/molecules27123858 (PMC9228897; doi:10.3390/molecules27123858)
Supplement: Supplementary file 1 [file molecules-27-03858-s001.zip › molecules-1760423-supplementary.pdf]

SUPPLEMENTARY DATA

Table S1: Primers used for RT-qPCR

Genes	Forward (5'-3′)	Reverse (3'-5')	Annealing temperatures	Product sizes (bp)
daf-16	ATCATCTTTCCGTCCCCG	TTGGAATTGCTGGAACCG	60	140
hsp-16.2	TGTTGGTGCAGTTGCTTCGAATC	TTCTCTTCGACGATTGCCTGTTG	60	117
hsp-70	ACCCTTCGTTGGATGGAACG	GCATCCGGAACCTGATTGGGC	60	135
sod-3	ATCTACTGCTCGCAGTGCTT	TTTCATGGCTGATTACAGGTT	60	128
gst-4	CGTTTTCTATGGAAGTGACGC	TCAGCCCAAGTCAATGAGTC	60	150
ctl-1	CCAAACAGCCACCCAAATCA	CGGATACCGTACTCGTGATGAT	60	185
Y45F10 D.4	CGAGAACCCGCGAAATGTCGGA	CGGTTGCCAGGGAAGATGATGAGGC	60	136

Table S2: Statistics of lifespan of N2 *C. elegans*

Groups	Number of worms	Median lifespan (Days)	Logrank (Mantel-Cox) P-value vs Control
DMSO	30	22.5	-
OP50	30	24	0.5997
5 μg/mL	30	29	0.0005
25 μg/mL	30	28	0.0110
50 μg/mL	30	29	0.0024

Table S3: Heat stress survival of N2 *C. elegans* statistics

Group	Number of worms	Median lifespan (Days)	Logrank (Mantel-Cox) P-value vs Control
DMSO	30	6	-
AE	30	10	0.0095

Table S4: *C. elegans* strains oxidative stress statistics

Strain	Group	Number of worms	Median lifespan (Days)	Logrank (Mantel-Cox) P-value vs Control
N2	DMSO	12	2	-
	AE	12	4	0.0057
daf-2	DMSO	12	5.5	-
	AE	12	6.5	0.0765
daf-16	DMSO	12	4	-
	AE	12	4.5	0.1767
skn-1	DMSO	12	2	-
	AE	12	2	0.4330
hsf-1	DMSO	12	4.5	-
	AE	12	4	0.2299
